# Modifications of Au Nanoparticle-Functionalized Graphene for Sensitive Detection of Sulfanilamide

**DOI:** 10.3390/s18030846

**Published:** 2018-03-13

**Authors:** Bao-Shan He, Xiao-Hai Yan

**Affiliations:** School of Food Science and Technology, Henan Key Laboratory of Cereal and Oil Food Safety Inspection and Control, Henan University of Technology, Zhengzhou 450001, China; taotao5510@126.com

**Keywords:** electrochemical sensor, Au nanoparticles, graphene, sulfanilamide

## Abstract

In this paper, we present a simple and feasible electrochemical sensor based on Au nanoparticle-functionalized graphene for the determination of sulfanilamide. Au nanoparticles were deposited on graphene, which acted as a platform to prepare excellent nanocomposites. Attributed to the graphene’s large surface area and the Au nanoparticles’ strong conductivity, many sulfanilamide molecules were enriched on the sensor surface and the signal response became more sensitive. Under the optimal conditions, the electrochemical sensors could be used for the efficient detection of sulfanilamide. Good linearity was observed in the range of 0.1–1000 μmol·L^−1^ and the detection limit was 0.011 μmol·L^−1^. Most importantly, the Au nanoparticle-functionalized graphene-modified electrode could be successfully applied for the detection of sulfanilamide in animal meat, and exhibited good stability, acceptable recovery, and offered a promising platform for point-of-care detecting in real samples.

## 1. Introduction

Sulfonamides (SAs) are one of the oldest classes of antibiotic drugs with widespread applications in prophylactic, therapeutic, and growth promoting [1,2] treatments, thanks to their low toxicity and high efficiency against bacterial diseases [3,4,5,6]. Recently, however, due to the abuse of SAs, their residues have become a considerable concern. It has reported that long-term use can produce certain side effects, such as emiction [6], hemopoiesis turbulence [6,7], allergic hypersensitivity reactions in humans, and carcinogen resulting in hypothyroid [8]. In response to the dangers of SAs, the maximum residue level (MRL) of SAs was established as 100 µg/kg [9] in the European Union.

To date, microbiological methods [10], immunoassays [11,12], spectrophotometry [13], chromatography [14,15], and liquid chromatography tandem mass spectrometry (LC-MS/MS) [16,17,18] are widely used in the detection of SAs. All of these methods are restricted with costly instruments, complicated operation processes, and time-consuming detection processes. However, electrochemical methods [19,20] have seen rapid development owing to their superior sensitivity, high accuracy, and suitability for point-of-care testing. For example, Fe_3_O_4_/Gr/GCE electrochemical sensors [19] and MIPs/QDs@SiO_2_ fluorescence sensors [21] have been reported for the detection of sulfanilamide. Obviously, electrochemical sensors have a lower detection limit. Hence, it is necessary to develop electrochemical methods for the detection of sulfanilamide.

Graphene (Gr), with a one-atom-thick honeycomb structure, has been widely used in many fields owing to its outstanding properties such as high surface area, low cost, good stability, good conductivity, and biocompatibility [22,23,24,25]. Thanks to its large surface and distinctive structure, Gr can be made into excellent nanocomposites in combination with a wide variety of nanomaterials. Au nanoparticles (AuNPs) are famous for their superior properties, such as ease of functionalization and synthesis, chemical stability, and good biocompatibility and conductivity [26,27]. Because of these unique properties, AuNPs can be easily utilized as an excellent functionalized material.

**Scheme 1:**
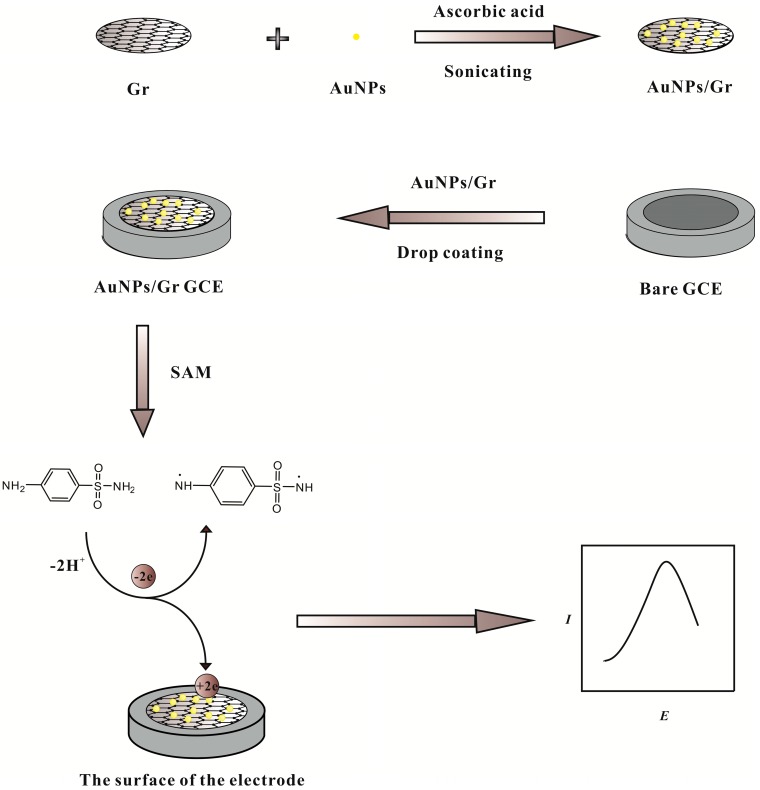
Schematic diagram of the sulfanilamide sensor.

In this work, a simple, feasible, and convenient electrochemical sensor for detecting sulfanilamide based on AuNP-functionalized Gr was constructed. Gr provided a large platform and the introduction of AuNPs raised its stability and maintained its inherent performances, for example, its large surface area and excellent conductivity. The AuNPs/Gr promoted electron transfer in the electropolymerization oxidation of sulfanilamide as electron transfer mediators when analytes were enriched on the surface of the electrode (Scheme 1). According to the changes of the differential pulse voltammetry response current, the detection of sulfanilamide could be achieved.

## 2. Experimental

### 2.1. Materials and Reagents

All chemicals were of at least analytical grade and were used without further purification. Gr was purchased from Ningbo Institute of Industrial Technology. *N*,*N*-Dimethyl formamide (DMF), Sulfanilamide (SAM), and Ascorbic Acid were obtained from Tianjin Kemiou Chemical Reagent Co., Ltd. (Tianjin, China). Chloroauric Acid (HAuCl_4_) was provided by Shanghai Aladdin Biochemical Technology Co., Ltd. (Shanghai, China). Potassium ferricyanide (K_3_[Fe(CN)_6_]), potassium ferrocyanide (K_4_[Fe(CN)_6_]), and reagents for preparing Britton-Robinson (BR) buffer solution (HBO_3_, H_3_PO_4_, H_2_SO_4_, HNO_3_) were purchased from Luoyang Chemical Reagent Factory (Luoyang, China). AuNPs/Gr nanocomposites were dissolved in DMF. SAM solutions were prepared in 0.04 mol·L^−1^ Britton-Robinson (BR) buffer solution. Any other solvent used was double distilled water, if not clearly indicated otherwise. 

### 2.2. Instruments

The morphology of materials was investigated via transmission electron microscopy (TEM) on an HT7700 (Hitachi, Japan) and energy dispersive X-ray (EDX) spectroscopy. Ultraviolet spectrophotometer (UV-vis) absorption spectra were recorded using a UV-6100s Double beam spectrophotometer (Mapada, Shanghai, China). Differential pulse voltammetry (DPV), cyclic voltammetry (CV), and electrochemical impedance spectroscopy (EIS) measurements were carried out using a CHI660E electrochemical workstation (Shanghai Chen Hua Instrument Co., Ltd., Shanghai, China).

### 2.3. Synthesis of AuNPs/Gr Nanocomposites

AuNPs/Gr nanocomposites were prepared according to a previously reported method [28]. At room temperature, 800 μL of 5 mmol·L^−1^ chloroauric acid solution was added to a glass beaker containing 400 μL of 1.0 g·L^−1^ Gr. The mixture was mixed evenly after the addition of 7.8 mL double distilled water and the homogeneous suspension was formed by sonication for 5 min. Meanwhile, 1 mL ascorbic acid solution (0.1 mol·L^−1^) was slowly added to the abovementioned glass beaker and kept for 2 days after 40 min of treatment. Centrifugation and washing were executed to remove most of the matrices, including ascorbic acid. Lastly, products were collected for further use after drying.

### 2.4. Fabrication of the AuNPs/Gr/GCE

A conventional three-electrode cell was used containing a platinum wire as a counter electrode, Ag/AgCl (saturated KCl) as the reference electrode, and a modified glassy carbon electrode (GCE, Φ = 3 mm) as the working electrode. The bare GCE was firstly polished with 0.05 mm alumina powder, followed by successive sonication in (1:1) HNO_3_, ethanol, and water to obtain a cleaned GCE surface. The cleaned GCE was activated by CV scanning in a 0.5 mol·L^−1^ H_2_SO_4_ solution before use. AuNPs/Gr (1.0 g·L^−1^) suspensions were prepared by ultrasonically dispersing 1 mg AuNPs/Gr nanocomposites in 1 mL DMF. Then, 2 μL as-prepared AuNPs/Gr (1.0 g·L^−1^) suspensions were dropped onto cleaned GCE and dried for measuring CV and EIS, which were conducted in 5 mM [Fe(CN)_6_]^3−^ solution containing 0.10 mol·L^−1^ KCl and 10 mM [Fe(CN)_6_]^3−/4−^ solution containing 0.10 mol·L^−1^ KCl as a redox probe, respectively [29]. The preparation of Gr/GCE was the same as the abovementioned process. All of the experiments were carried out at room temperature.

## 3. Results and Discussion

### 3.1. Characterization of AuNPs/Gr

**Figure 1:**
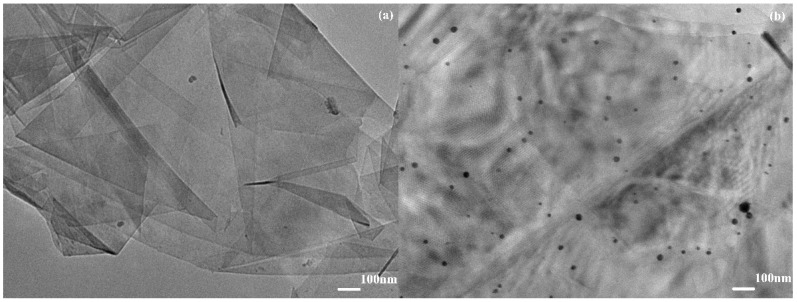
TEM images of Gr (**a**) and Au nanoparticles (AuNPs)/Gr (**b**).

**Figure 2:**
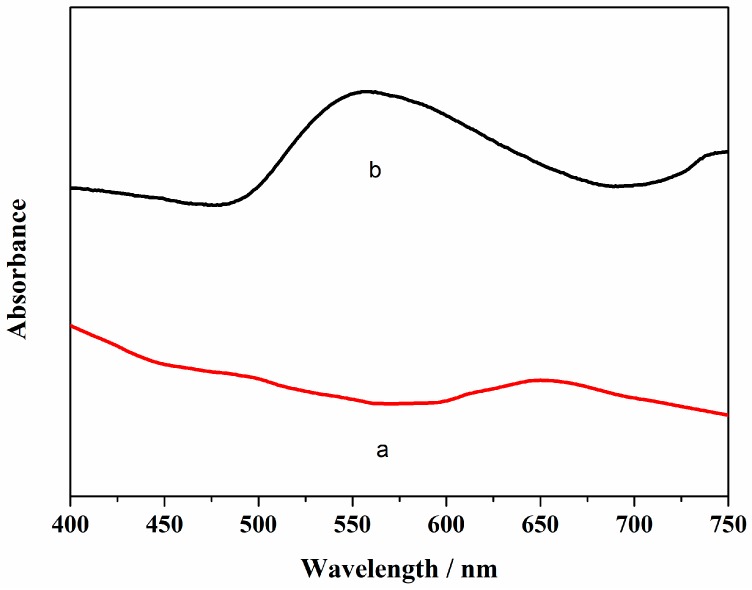
UV-vis absorption spectra of (**a**) Gr and (**b**) AuNPs/Gr.

Transmission electron microscopy (TEM) images of AuNPs/Gr in Figure 1 were used to support the successful attachment of AuNPs onto Gr. As illustrated in Figure 1a, the Gr exhibited a thin, flaky, and folding structure that demonstrated a large surface area. Figure 1b shows that a number of small black particles were evenly dispersed on the Gr surface, showing the successful preparation of the AuNPs/Gr nanocomposites. The UV-vis spectra of Gr and AuNPs/Gr were recorded. As shown in Figure 2a, no absorption peak was observed for pure Gr. After AuNPs were mixed with Gr, Figure 2b shows an obvious absorption peak at approximately 550 nm wavelength, like that of pure AuNPs. Both UV-vis spectra and TEM demonstrated that AuNPs/Gr nanocomposites were constructed successfully. All of the abovementioned results were consistent with the previous literature [30].

**Figure 3:**
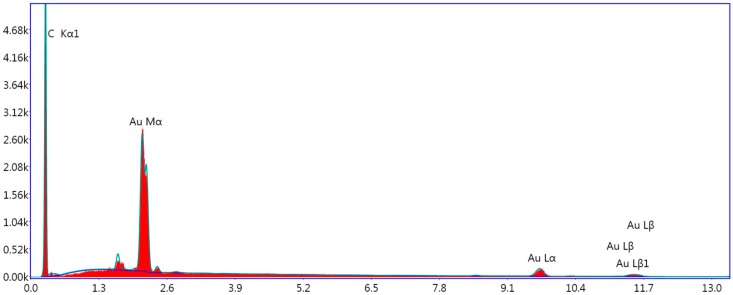
Energy dispersive X-ray EDX spectrum of the AuNPs/Gr nanocomposites.

To further verify the successful synthesis of AuNPs/Gr nanocomposites, the EDX spectrum of AuNPs/Gr nanocomposites was measured to determine elemental compositions. In Figure 3, the peaks corresponding to Au and C elements are clearly observed, indicating the presence of AuNPs and Gr and the validity of the above conclusion.

### 3.2. Surface Morphology of the Modified Electrodes

**Figure 4:**
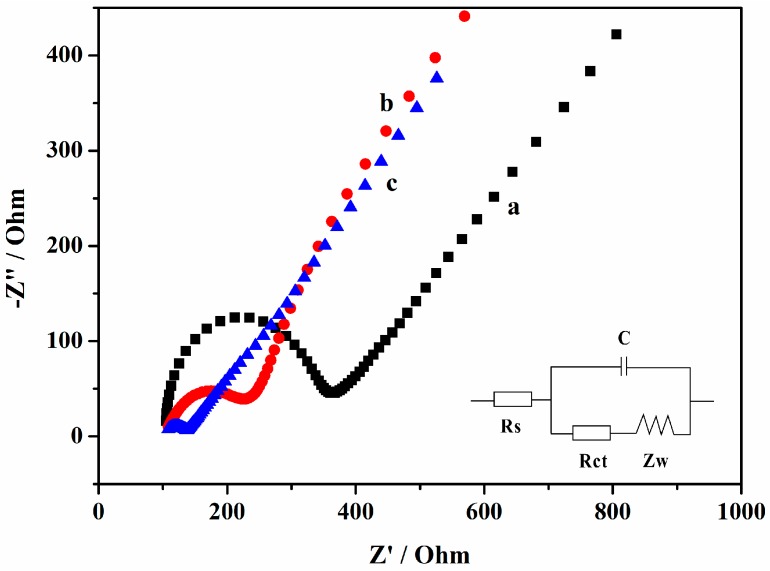
Electrochemical impedance spectroscopy (EIS) measurements of different modified glassy carbon electrodes (GCEs) in 0.10 mol·L^−1^ KCl aqueous solution containing 10 mM [Fe(CN)_6_]^3−/4−^: (a) bare GCE; (b) Gr/GCE; (c) AuNPs/Gr/GCE. The inset is the equivalent electric circuit of impedance.

The characterization of the electron transfer properties of the different modified electrodes is always carried out using EIS, which is measured in [Fe(CN)_6_]^3−/4−^ solution [31]. Ferrocyanide/ferricyanide act as strong redox probes, which are used to judge if the electrode surface modified successfully by whether the electron transfer resistance (Rct) changed as expected. The larger the Rct is, the larger the Nyquist diagram semicircular diameter is. Figure 4 shows the Rct of the GCE, Gr/GCE, and AuNPs/Gr/GCE, respectively. The Rcts were obtained using the equivalent circuit (the inset of Figure 3). The bare GCE exhibited relatively good conductivity and the Rct was approximately 243.5 Ω (curve a). Upon the modification of Gr, the Rct became 75.13 Ω (curve b) and the diameter was obviously decreased, which was attributed to the superior conductivity of Gr which thus increased the electron transfer rate. After modifying AuNPs/Gr, the diameter of the semicircle was further decreased and the Rct was approximately 25.42 Ω (curve c), implying that the combination of Gr and AuNPs provided stronger conductivity, a larger electrochemically active surface area, and made more electrons reach the surface of the GCE.

### 3.3. Electrochemical Response of SAM on AuNPs/Gr/GCE

**Figure 5:**
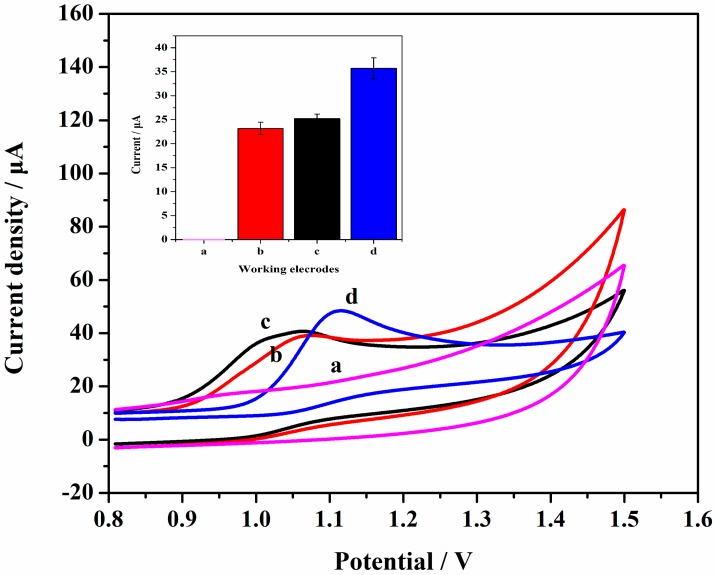
Cyclic voltammetry (CV) curves of (a) bare GCE without Sulfanilamide (SAM); (b) bare GCE; (c) Gr/GCE; and (d) AuNPs/Gr/GCE in 1.0 × 10^−3^ mol·L^−1^ SAM (pH = 2.0) .The inset is the quantification of peaks appearing on the voltammogram. The potential sweep rate was 100 mV·s^−1^.

As shown in Figure 5, no oxidation peak was observed when using the GCE to scan in the absence of SAM (curve a). Also in Figure 5, the bare GCE showed a significant oxidation peak in the presence of SAM and the oxidation peak current value was 23.19 μA at about 1.064 V (curve b). The oxidation peak current value of Gr/GCE increased slightly to 25.22 μA and the electric potential was about 1.076 V (curve c). Compared with the Gr/GCE, the oxidation peak current value of AuNPs/Gr/GCE increased significantly to 35.71 μA at about 1.115 V (curve d). Figure 5 indicates that unmodified and modified electrodes generated an obvious signal response in the presence of SAM (curves b, c, and d to curve a). The modification of Gr increased the activity area and conductivity of the electrode (curve c to curve b). The significant increase of the oxidation peak (curve d to curve c) indicated that the introduction of AuNPs further enhanced conductivity.

### 3.4. Effects of Different Scanning Rates

**Figure 6:**
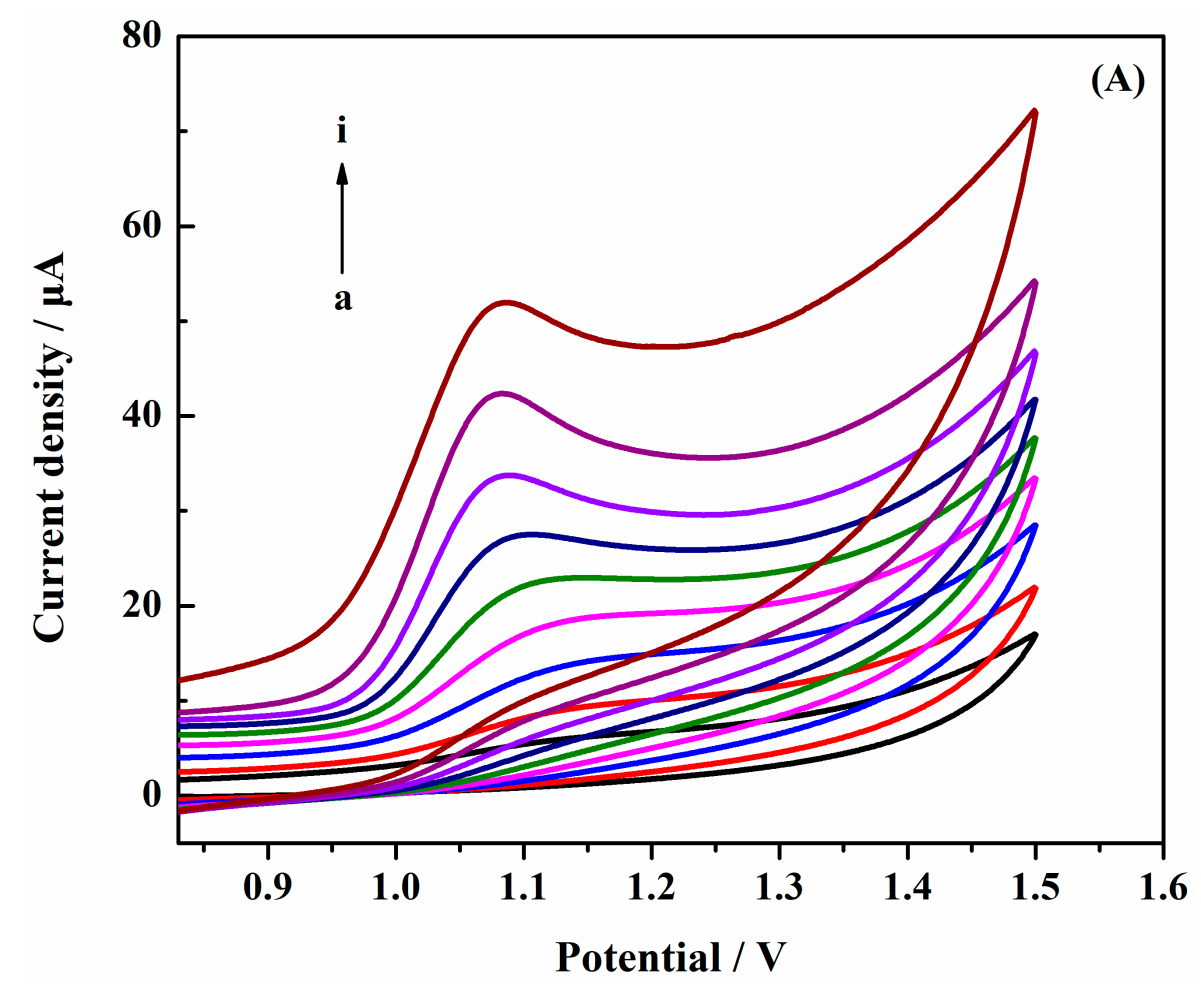

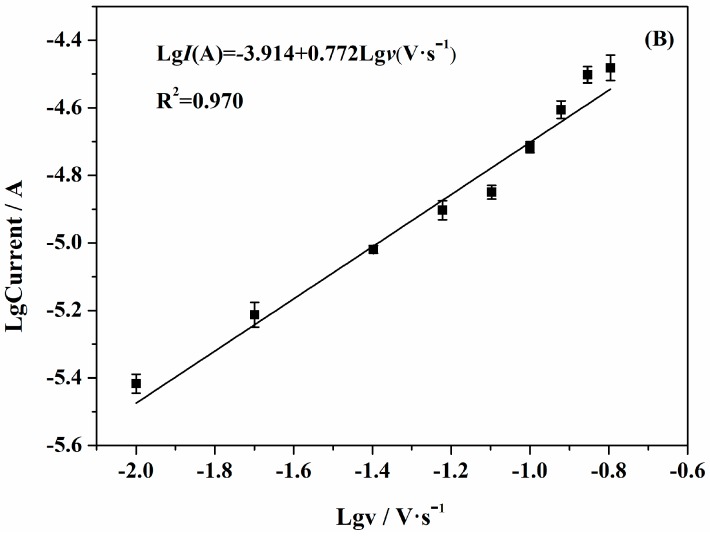
(**A**) CVs of AuNPs/Gr/GCE at different scan rates: (a) 10; (b) 20; (c) 40; (d) 60; (e) 80; (f) 100; (g) 120; (h) 140; (i) 160 mV·s^−1^; (**B**) Relationship of the logarithm of oxidation peak current versus the logarithm of scan rates.

Figure 6a shows the CVs of different scan rates in the determination of 1.0 × 10^−3^ mol·L^−1^ SAM at the surface of AuNPs/Gr/GCE. As shown in Figure 6b, the logarithm of oxidation peak current value increased linearly as the logarithm of scan rates increased, and the linear regression equation was: Lg*I*(A) = − 3.914 + 0.772 Lg*v*(V·s^−1^) (R^2^ = 0.970). The slope was 0.772, illustrating an adsorption-controlled process of SAM on AuNPs/Gr/GCE. Therefore, in order to prevent excessive charging current and obtain a better peak shape, we chose a scanning rate of 100 mV·s^−1^.

### 3.5. Influence of pH

**Figure 7:**
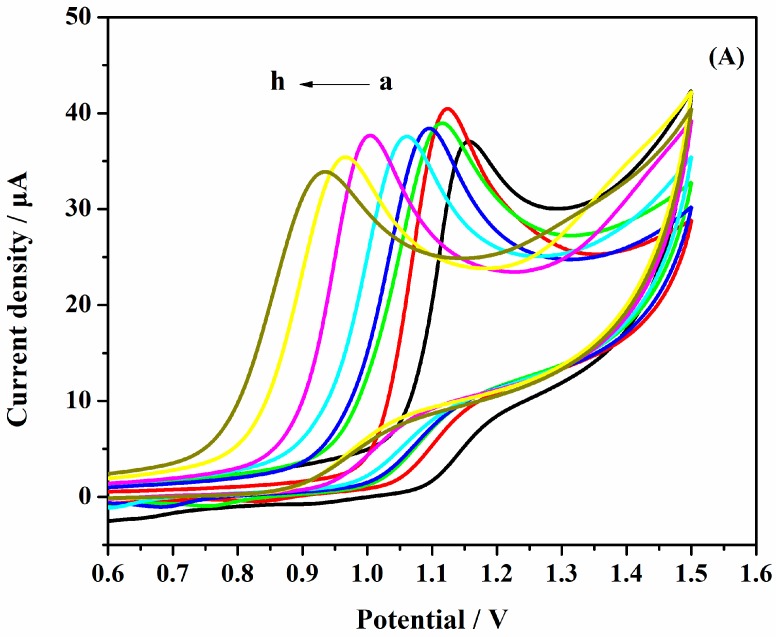

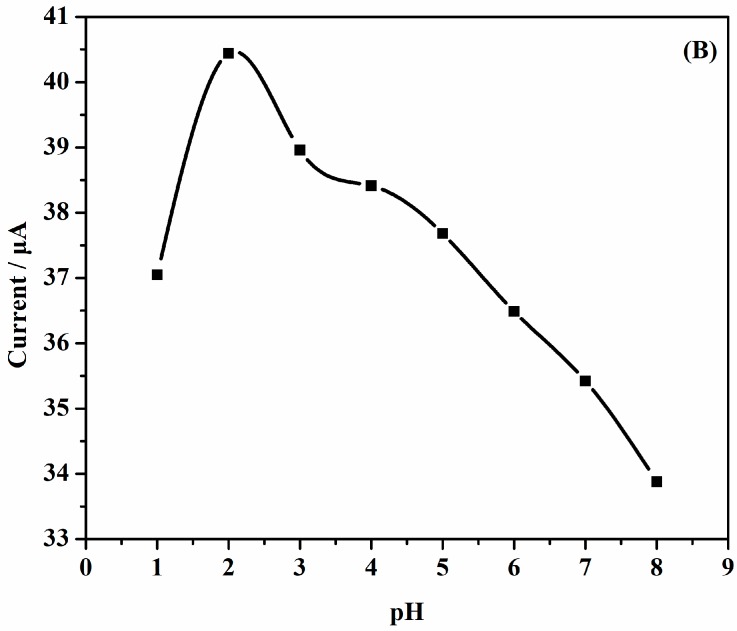
(**A**) CVs of a solution of 1.0 × 10^−3^ mol·L^−1^ SAM AuNPs/Gr/GCE at various buffered pHs: (a) 1.0; (b) 2.0; (c) 3.0; (d) 4.0; (e) 5.0; (f) 6.0; (g) 7.0; (h) 8.0; (**B**) Effects of pH and the oxidation peak current of 1.0 × 10^−3^ mol·L^−1^ SAM.

The effects of pH on the performance of AuNPs/Gr/GCE were studied systematically. Figure 7a depicts the CV curves at different pHs. The potential was shifted to less values with increasing pH, which may be due to the effect of protons. Figure 7b illustrates the relationship diagram of the oxidation peak current versus pHs, and it was easy to find that the highest value of pH was 2.0. As the pH value became larger or smaller, other oxidation peak current values decreased. It could thus be concluded that the optimal pH value was 2.0.

### 3.6. Effects of Different AuNPs/Gr Modification Volume

**Figure 8:**
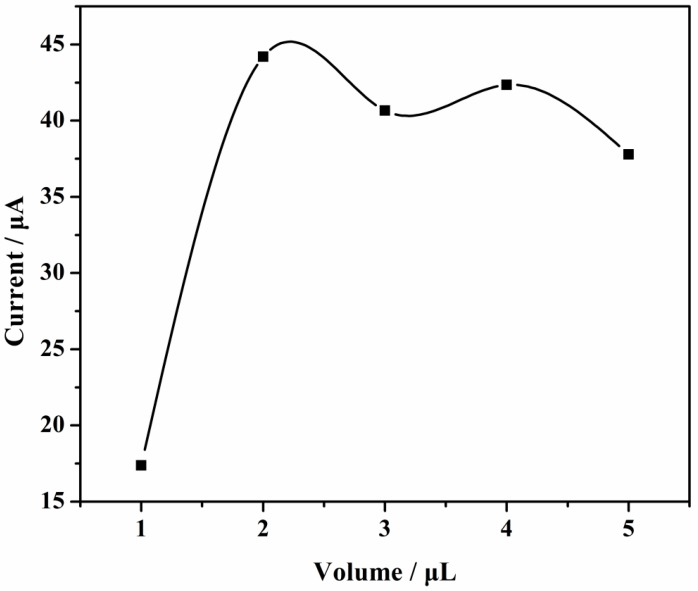
Relationship of the oxidation peak current versus volume of modification.

As can be seen from Figure 8, when the modification volume was 2 μL, the oxidation peak current was the highest and the electrochemical response was the strongest. Therefore, 2 μL was chosen as the best volume of modification.

### 3.7. Determination of SAM

**Figure 9:**
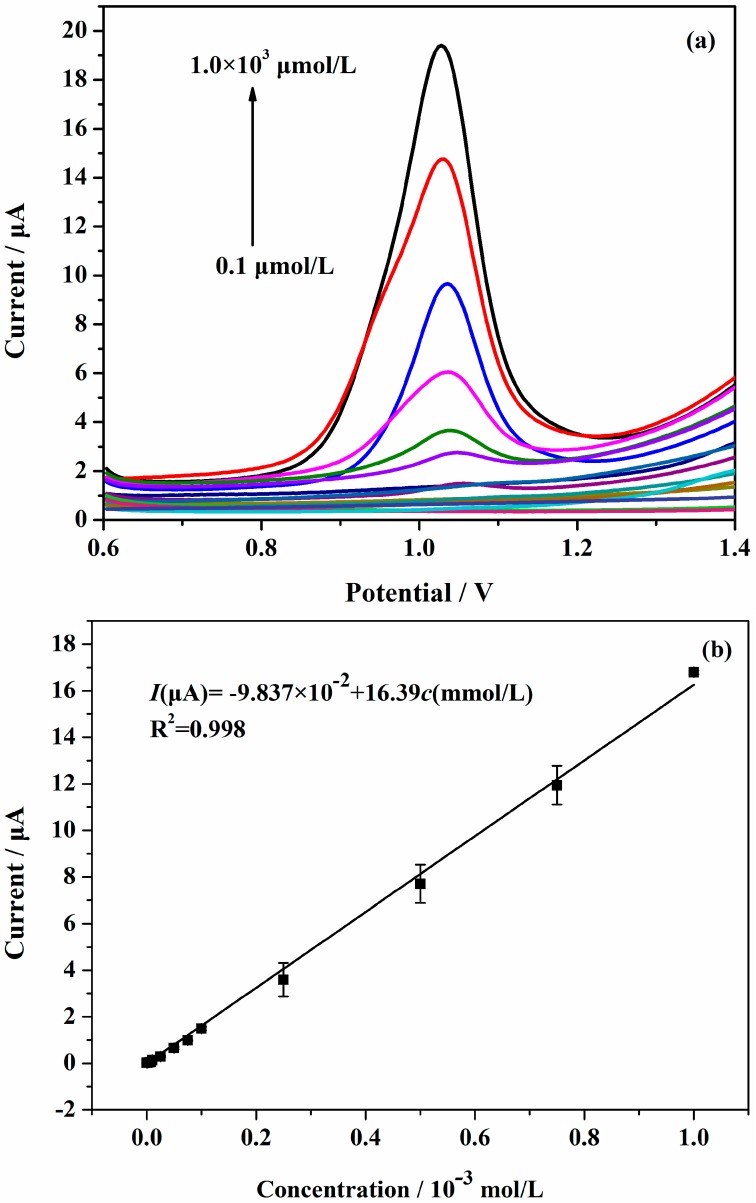
(**a**) Differential pulse voltammetry (DPV) measurements of different concentrations of SAM in 0.04 mol·L^−1^ Britton-Robinson (BR) buffer solution (pH 2.0) using 2 μL AuNPs/Gr/GCE; (**b**) The plots of linear relationship between SAM concentration and oxidation peak current: 0.1 to 1000 μmol·L^−1^. The potential sweep rate was 100 mV·s^−1^. (Amplitude: 0. 05 V, pulse width: 0.2 s, sampling width: 0.0167 s, pulse period: 0.5 s).

Under the optimal conditions, the standard curve was determined by using AuNPs/Gr/GCE to measure the oxidation peak current of a series of different concentrations of SAM solution. Based on the differential pulse voltammetry (DPV) method, as shown in Figure 9a, as the concentration of the SAM solution increased, the DPV current also increased. Figure 9b shows a good linearity of the DPV current versus SAM concentrations in the range of 0.1–1000 μmol·L^−1^. The linear regression equation was: *I* (μA) = − 9.837 × 10^−2^ + 16.39*c* (mmol·L^−1^) (R^2^ = 0.998). From the plot, the calculated limit of detection (LOD) was 0.011 μmol·L^−1^ on the basis of the 3SD/*n* method, where ‘SD’ is the standard deviation of the blank solution and ‘*n*’ is the slope of the standard curve. The experimental result indicated that our proposed method possessed a wider dynamic range and a lower limit of detection relative to previous methods (Table 1).

### 3.8. Interferences Study

To evaluate the effects of interfering substances on the detection of SAM, CV was performed in 1.0 × 10^−3^ mol·L^−1^ SAM solution containing interference species. Specifically, 200-fold of glucose and sucrose, and 300-fold of NaCl, KCl, CaCl_2_, and MgSO_4_ were added into the SAM solutions, respectively. It was found that 300-fold of NaCl, KCl, CaCl_2_, MgSO_4_ had little interference effect on the detection of SAM, but 200-fold of glucose and sucrose had a great impact. As shown in Table 2, only 50-fold of sucrose and glucose led to approximately ±5% relative error in the assay. Therefore, in the real sample testing, the effects of a high concentration of glucose and sucrose needed to be considered.

### 3.9. Repeatability and Stability

To evaluate the repeatability and stability, the corresponding experiments were studied. Under optimized conditions, SAM of 1.0 × 10^−3^ mol·L^−1^ was continuously scanned 12 times. In this work, the change of oxidation peak current that occurred the first three times was small, but afterwards it changed obviously. The reason for this may be the surface passivation of AuNPs/Gr/GCE under acidic conditions, which hindered the electron transfer to the surface of AuNPs/Gr/GCE. The AuNPs/Gr/GCEs were observed for 0–15 h, respectively. The peak current of newly prepared AuNPs/Gr/GCE was 32.23 μA. After 15 h, the peak current was 29.01 μA with a reduction of 9.71% and the oxidation peak potential was almost unchanged. Therefore, AuNPs/Gr/GCE had high electrocatalytic activity and good stability.

### 3.10. Application of Sensor in a Real Sample

To prove the feasibility of the AuNPs/Gr/GCE in real sample, the calibration experiment was studied. Pork extract was selected as the sample, and the content and recovery of SAM in the diluted sample solutions were determined via the standard adding method. The established standard curve equation in the above experiment was used to calculate the content of SAM. The results are shown in Table 3. In the three parallel experiments the average recoveries ranged from 91.22% to 96.67%, thus indicating that the sensor could successfully detect SAM in a real sample.

## 4. Conclusions

A simple and feasible electrochemical sensor based on AuNPs/Gr nanocomposites was provided for detecting SAM. The AuNPs/Gr nanocomposites have a better performance compared with pure Gr for the detection of SAM owing to their high conductivity and large surface area. Furthermore, the prepared AuNPs/Gr/GCE possessed a certain stability, good selectivity, and acceptable recovery abilities. Based on the abovementioned characteristics, it could be promising in the practical application of detecting in a real sample.

## Figures and Tables

**Table 1:** Comparison of SAM determinations using the proposed and reference methods.

Method	Electrode	Linear Range (μmol·L^−1^)	Limit of Detection	Reference
*i-t*	Fe_3_O_4_/Gr/GCE	0.5–110	0.05	[19]
CV	Carboxyl/DMF/MWCNTs/GCE ^a^	1.0–100	0.5	[20]
FL ^b^	MIPs/QDs@SiO_2_^c^	2.0–30	0.17	[21]
DPV	Py/MIP PGE ^d^	0.05–1.1, 1.1–48	0.02	[32]
DPV	Gr/AuNPs/GCE	0.1–1000	0.011	This work

^a^ Carboxyl/*N*,*N*-dimethylformamide/Multi-walled carbon/GCE; ^b^ Fluorescence; ^c^ Molecularly imprinted polymers/Quantum dots@SiO_2_; ^d^ Pyrrole/Molecularly imprinted polymer pencil graphite electrode.

**Table 2:** Effects of interfering substances on the determination of SAM solution of 1 × 10^−3^ mol·L^−1^ by AuNPs/Gr/GCE.

Solution (mol·L^−1^)	Current (μA)	Rate of Change (%)
1.0 × 10^−3^ SAM	26.18	3.44
After addition of 50-fold glucose	25.38	
1.0 × 10^−3^ SAM	22.28	3.00
After addition of 50-fold sucrose	22.95	
1.0 × 10^−3^ SAM	27.69	2.60
After addition of 300-fold NaCl	28.41	
1.0 × 10^−3^ SAM	29.44	4.55
After addition of 300-fold KCl	30.78	
1.0 × 10^−3^ SAM	28.48	3.55
After addition of 300-fold CaCl_2_	29.49	
1.0 × 10^−3^ SAM	28.46	4.56
After addition of 300-fold MgSO_4_	29.76	

**Table 3:** Recovery experimental results of the determination of SAM in a real sample.

Sample	Added (10^−5^ mol·L^−1^)	Found (10^−5^ mol·L^−1^)	Recovery (%)	Average Recovery (%)
SAM	3.0	2.69	89.67	91.22
		2.74	91.33	
		2.78	92.67	
	6.0	5.43	90.50	96.67
		5.85	97.50	
		6.12	102.00	
	9.0	7.73	85.89	94.67
		8.76	97.33	
		9.07	100.78	
